# Vitamin D is involved in regulating limbic epileptogenesis

**DOI:** 10.1093/braincomms/fcag049

**Published:** 2026-02-16

**Authors:** Shuya Qi, Mingyue Chen, Lihang Wei, Yang Wang, Xixia Liu, Bingyan Wang, Yuanyuan Liu, Guohe Tan

**Affiliations:** Department of Human Anatomy, Institute of Neuroscience and Guangxi Key Laboratory of Brain Science, School of Basic Medical Sciences, Guangxi Medical University, Nanning, Guangxi 530021, China; Key Laboratory of Longevity and Aging-Related Diseases of Chinese Ministry of Education, Guangxi Medical University, Nanning, Guangxi 530021, China; Health Commission Key Laboratory of Basic Research on Brain Function and Disease, Key Laboratory of Human Development and Disease Research by Education Department of Guangxi Zhuang Autonomous Region, Nanning, Guangxi 530021, China; Department of Human Anatomy, Institute of Neuroscience and Guangxi Key Laboratory of Brain Science, School of Basic Medical Sciences, Guangxi Medical University, Nanning, Guangxi 530021, China; Key Laboratory of Longevity and Aging-Related Diseases of Chinese Ministry of Education, Guangxi Medical University, Nanning, Guangxi 530021, China; Health Commission Key Laboratory of Basic Research on Brain Function and Disease, Key Laboratory of Human Development and Disease Research by Education Department of Guangxi Zhuang Autonomous Region, Nanning, Guangxi 530021, China; Department of Human Anatomy, Institute of Neuroscience and Guangxi Key Laboratory of Brain Science, School of Basic Medical Sciences, Guangxi Medical University, Nanning, Guangxi 530021, China; Key Laboratory of Longevity and Aging-Related Diseases of Chinese Ministry of Education, Guangxi Medical University, Nanning, Guangxi 530021, China; Health Commission Key Laboratory of Basic Research on Brain Function and Disease, Key Laboratory of Human Development and Disease Research by Education Department of Guangxi Zhuang Autonomous Region, Nanning, Guangxi 530021, China; Collaborative Innovation Centre of Regenerative Medicine and Medical BioResource Development and Application Co-Constructed by the Province and Ministry, Guangxi Key Laboratory of Regenerative Medicine, Nanning, Guangxi 530021, China; Key Laboratory of Longevity and Aging-Related Diseases of Chinese Ministry of Education, Guangxi Medical University, Nanning, Guangxi 530021, China; Collaborative Innovation Centre of Regenerative Medicine and Medical BioResource Development and Application Co-Constructed by the Province and Ministry, Guangxi Key Laboratory of Regenerative Medicine, Nanning, Guangxi 530021, China; Department of Rehabilitation, The People's Hospital of Guangxi Zhuang Autonomous Region, Nanning, Guangxi 530213, China; Department of Human Anatomy, Institute of Neuroscience and Guangxi Key Laboratory of Brain Science, School of Basic Medical Sciences, Guangxi Medical University, Nanning, Guangxi 530021, China; Key Laboratory of Longevity and Aging-Related Diseases of Chinese Ministry of Education, Guangxi Medical University, Nanning, Guangxi 530021, China; Health Commission Key Laboratory of Basic Research on Brain Function and Disease, Key Laboratory of Human Development and Disease Research by Education Department of Guangxi Zhuang Autonomous Region, Nanning, Guangxi 530021, China; Department of Human Anatomy, Institute of Neuroscience and Guangxi Key Laboratory of Brain Science, School of Basic Medical Sciences, Guangxi Medical University, Nanning, Guangxi 530021, China; Health Commission Key Laboratory of Basic Research on Brain Function and Disease, Key Laboratory of Human Development and Disease Research by Education Department of Guangxi Zhuang Autonomous Region, Nanning, Guangxi 530021, China; Department of Human Anatomy, Institute of Neuroscience and Guangxi Key Laboratory of Brain Science, School of Basic Medical Sciences, Guangxi Medical University, Nanning, Guangxi 530021, China; Key Laboratory of Longevity and Aging-Related Diseases of Chinese Ministry of Education, Guangxi Medical University, Nanning, Guangxi 530021, China; Health Commission Key Laboratory of Basic Research on Brain Function and Disease, Key Laboratory of Human Development and Disease Research by Education Department of Guangxi Zhuang Autonomous Region, Nanning, Guangxi 530021, China; Collaborative Innovation Centre of Regenerative Medicine and Medical BioResource Development and Application Co-Constructed by the Province and Ministry, Guangxi Key Laboratory of Regenerative Medicine, Nanning, Guangxi 530021, China

**Keywords:** vitamin D, vitamin D receptor, temporal lobe epilepsy, limbic epilepsy, kindling model

## Abstract

Limbic epilepsy is a common brain disorder associated with dysregulation of micronutrients, of which vitamin D plays an important role in neuronal survival, neurotransmitter homeostasis and synaptic transmission. However, the *in vivo* contribution of vitamin D to epileptogenesis remains unclear. In this study, we observed a significant increase in vitamin D receptor (VDR) mRNA level at 6–12 h after seizure onset in four distinct classic epilepsy models. Vitamin D receptor protein expression also increased following pilocarpine-induced seizures, particularly in the hippocampal granule cell layer. Subsequently, we found that aberrant vitamin D dietary levels could accelerate kindling development. To our surprise, excessive vitamin D intake reduced the frequency of spontaneous seizures in the pilocarpine model, whereas vitamin D deficiency decreased mossy fibre sprouting—a hallmark of epileptogenesis. These findings provide new insights into the mechanisms underlying limbic epileptogenesis.

## Teaser

Qi, et al. find a significant increase in vitamin D receptor expression after seizure activity in multiple classic animal models of limbic epilepsy. Furthermore, aberrant vitamin D dietary levels affected kindling development, induced spontaneous mossy fibre sprouting. These findings help to understand the novel mechanisms of limbic epileptogenesis.

## Graphical Abstract

**Graphical Abstract:**
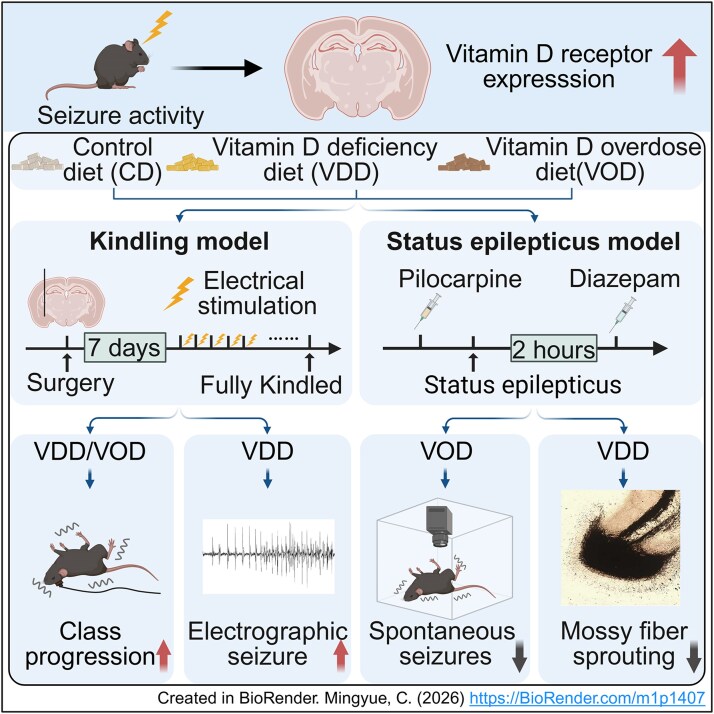

## Introduction

Epilepsy is one of the most common serious brain conditions characterized by excessive neuronal excitability and abnormal synchronous neuronal discharges. It affects individuals of all ages across the globe, with a worldwide prevalence of about 1–2%.^1^ Clinically, epilepsy is broadly classified into focal and generalized forms. Focal epilepsy typically arises from structural abnormalities such as trauma or cortical malformations and originates in discrete brain regions before possibly propagating to wider networks or causing secondary generalization. Generalized epilepsy is often of genetic or functional origin and involves bilateral, synchronous discharges primarily through the thalamo-cortical circuit.^2-4^ Focal epilepsy commonly manifests as unilateral motor or sensory symptoms or complex partial seizures with impaired awareness, whereas generalized epilepsy manifests as bilateral, synchronous absence or tonic–clonic seizures.^3,5^ Although antiepileptic drugs (AEDs) that target neuronal excitability and inhibitory transmission can reduce seizure frequency, they generally fail to prevent or reverse epileptogenesis. Furthermore, approximately one-third of patients develop drug-resistant epilepsy despite optimal pharmacological management,^6^ which imposes significant socioeconomic, psychosocial, physical and psychological burdens on patients, families and healthcare systems.^7^ Epileptogenesis involves various mechanisms, such as enhanced glutamatergic excitability and weakened GABAergic inhibition, that contribute to abnormal neuronal synchronization.^8^ Excessive excitatory activity drives synaptic potentiation^9^ and neuronal network reorganization, which in turn exacerbates seizure activity.^10,11^ Although these complex pathological changes may be linked to disruptions in brain micronutrient homeostasis, the underlying mechanisms are poorly understood.^12^

Given the complexity of epileptogenesis, identifying brain trace elements that could affect the pathophysiology of epilepsy is paramount. In this context, vitamin D (Vit.D) emerges as a potential element. Accumulating evidence indicates that Vit.D plays an important role in neuroplasticity, neuronal physiology and neurological diseases by regulating neurotransmitters, neuronal differentiation, axonal growth, calcium signalling and reactive oxygen in the brain.^13^ VDR is widely distributed in the brain and is expressed by neurons.^14^ A recent study has suggested that altered GABA levels are found in the brains of rodents fed with a Vit.D-deficiency diet,^15^ which prompts a potential role for Vit.D in the pathophysiology of epilepsy. Additionally, Vit.D has neuroprotective effects, and its levels in the body are affected by seasonal light exposure. Reduced sunlight exposure during winter results in diminished Vit.D production, which may potentially compromise its neuroprotective function.^16^ Recent epidemiological studies reported seizure exacerbations demonstrating seasonal variation, peaking in the winter months,^17^ while children with epilepsy also exhibit variations in their birth months.^18^ Evidence supports an association between Vit.D and epilepsy in humans. Vit.D deficiency during early childhood is a critical risk factor for seizures as evidenced by meta-analyses demonstrating a dose-dependent association.^19^ Clinical evidence has demonstrated that Vit.D-deficiency occurred in children with epilepsy *versus* healthy children,^16^ and inadequate Vit.D serum levels could be related to the difficulty in controlling seizures,^19-21^ indicating that epilepsy may be related to Vit.D. These findings suggest that Vit.D might be involved in the pathogenesis of epilepsy. However, few studies have reported on the direct role of Vit.D in the epileptogenesis of temporal lobe epilepsy (TLE). Whether Vit.D affects the pathological process of epilepsy has not been elucidated.

In this study, we addressed this knowledge gap by examining the involvement of Vit.D and VDR in limbic epileptogenesis using four male mouse models. First, we quantified VDR expression dynamics at multiple time points following seizure induction. Next, we investigated the effects of chronic low- and high-dose Vit.D diets on epileptogenesis, with mice subjected to kindling electrical stimulation through implanted stereotactic electrodes. We further evaluated the effects of Vit.D on spontaneous seizure occurrence and mossy fibre sprouting in the pilocarpine-induced mouse model. Finally, bioinformatic analysis was performed to explore potential molecular pathways through which Vit.D may affect epileptogenesis in limbic epilepsy.

## Material and methods

### Animals

All the animal experiments were performed in accordance with the guidelines of the Animal Management and Use Committee of the Experimental Animal Center of Guangxi Medical University. Male C57BL/6 mice (*Mus musculus*) were bred using a specific pathogen-free (SPF) system at the Experimental Animal Center of Guangxi Medical University (License No. SCXK Gui2003-0003). For the experiments, eight-week-old male mice (initial body weight 22–25 g) purchased from Beijing Vital River Laboratory Animal Technology Co., Ltd. (License No. SCXK Jing2015-0001) were used. The housing standard was 4–6 mice *per* cage subjected to 12 h light/dark cycles. The room temperature was maintained at 25 ± 2°C and the humidity was maintained at 50 ± 5%.

In each experiment, male C57BL/6 mice were randomly assigned to three dietary groups (*n* = 10 *per* group): control diet, Vit.D-overdose diet and Vit.D-deficiency diet. Healthy diets (Nantong Trofe Feed Technology Co., China) containing different amounts of Vit.D were used for mouse models. The control diet group mice were provided a diet (AIN93M) containing standard Vit.D content (Vitamin D3 of 2000 IU/kg). The Vit.D-overdose diet group mice were provided with a diet (LAD3000M) containing excessive amounts of Vit.D (Vitamin D3 of 3000 IU/kg), and the Vit.D-deficiency diet group mice were provided with a diet (TP2250M) with Vit.D-deficiency (Vitamin D3 < 25 IU/kg).^22^ This mouse model was established over 8 weeks.^23^ Diet for this model was limited to 4 g/mouse/day and was replenished every alternate day.

### Assessment of serum 25-hydroxyvitamin D [25(OH)D] levels

After 8 weeks of dietary feeding, some mice were euthanized and blood samples were collected via submandibular vein puncture. Serum samples were promptly separated and stored at −20°C for subsequent quantification of 25-hydroxyvitamin D [25(OH)D] levels using the Vitamin D ELISA Kit (Cat. No. E-EL-0012, Elabscience). Prior to assay, serum samples were thawed and analysed strictly in accordance with the manufacturer’s protocols. This kit exhibited a detection range of 6.25–400 ng/ml, a sensitivity of 3.75 ng/ml and universal reactivity. Optical density was measured, and the concentrations of 25(OH)D were calculated based on the provided standard curve.

### Severity of mice seizures

Seizure events were defined as regular spike clusters with a duration of ≥5 s, spike frequency of ≥2 Hz and amplitude at least three times that of the baseline EEG. In the electrically induced kindling model, electrical stimulation induced high-amplitude spike-waves in the EEG. The class of seizure activity was determined using the Racine criteria as follows: Stage 0, no twitches; Stage 1, visible local facial muscle twitches, including rhythmic blinking, chewing and whisker twitching; Stage 2, head nodding; Stage 3, visible unilateral forelimb clonus and convulsions; Stage 4, bilateral forelimb clonus, convulsions; and Stage 5, full-onset, body presenting dorsiflexion and rigidity, falling accompanied by bilateral rigidity of the hind limbs. Stages 1–3 were considered focal seizures, and Stages 4–5 were considered generalized seizures.^24^ According to the Racine scale, the seizure stage was scored by an investigator blinded to the group allocation.

### Chemically induced epilepsy model

#### Pilocarpine-induced status epilepticus model

Scopolamine hydrobromide trihydrate (1 mg/kg) was administered intraperitoneally (i.p.) 30 min before pilocarpine injection to reduce the peripheral cholinergic effects of pilocarpine. The mice received an injection of 350 mg/kg pilocarpine (i.p.), and the seizure activity was evaluated using the Racine criteria. Mice exhibiting persistent head nodding and chewing accompanied by forelimb or whole-body clonus after experiencing multiple episodes of Stage 4 or higher seizures were considered to have developed status epilepticus (SE). SE onset in mice was defined as the seizure activity after the first observation of Stage 4 or higher seizures that lasted at least 30 min. Mice that developed SE were administered diazepam (4 mg/kg, i.p.) 180 min post-SE onset to arrest the convulsions. Mice that did not develop SE were excluded from this study.

#### Kainic acid-induced SE model

Mice were administered kainic acid (KA) (20 mg/kg, i.p.) to induce seizures. The animals were observed, and any behavioural abnormalities were recorded. Mice that developed SE were included in the study and received diazepam (4 mg/kg, i.p.) to arrest the convulsions.

#### Pentylenetetrazol-induced acute epilepsy model

Pentylenetetrazol (PTZ) (60 mg/kg) was injected intraperitoneally, and mice were monitored for at least 30 min. Mice exhibiting Stage 5 seizures during this period were included in the study.

**Figure 1:**
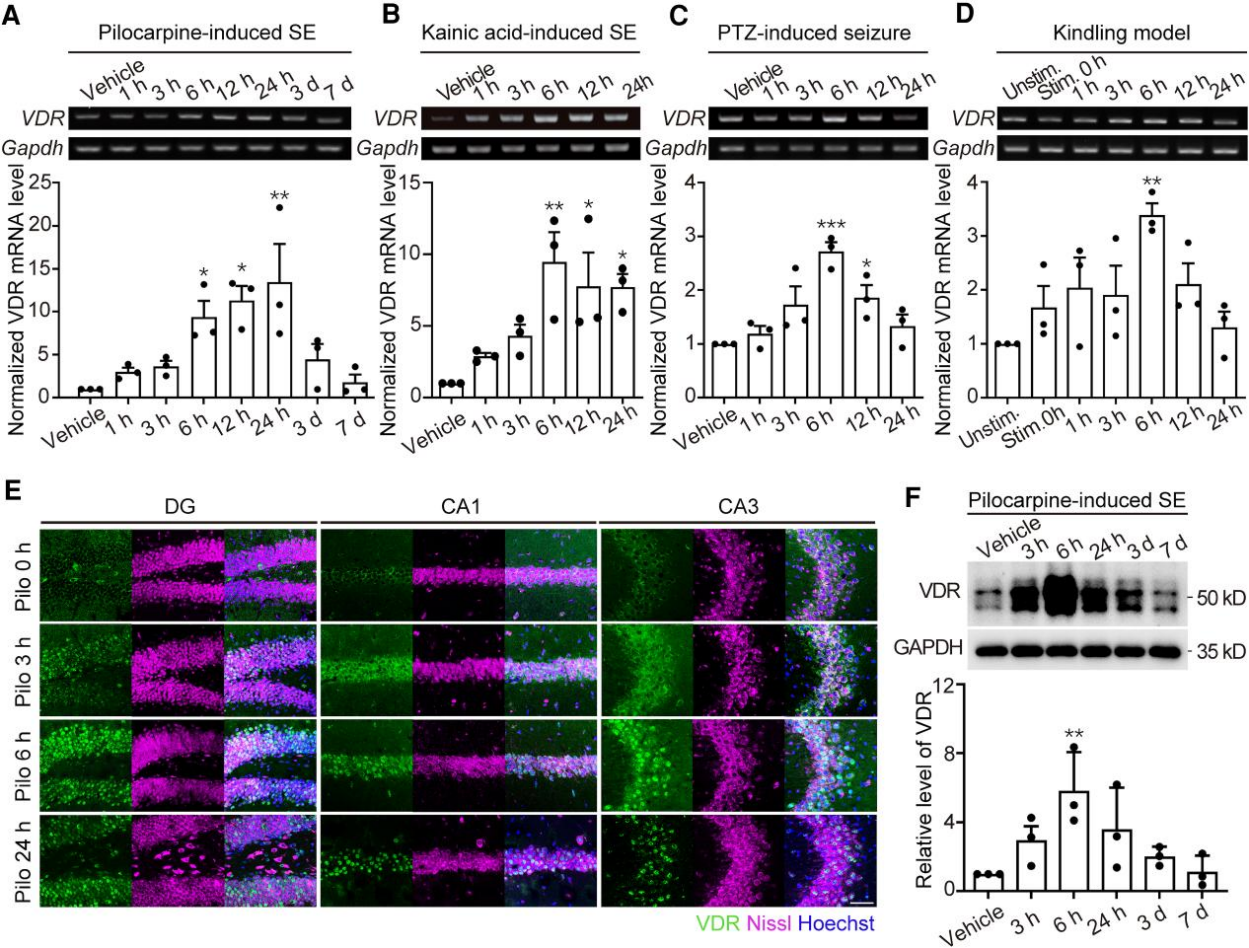
**VDR expression is upregulated in hippocampus following seizure activity in mouse models of TLE.** (**A–D**) PCR analysis reveals the temporal expression pattern of *VDR* mRNA at different time points after seizures in distinct epileptic models. **A**, Pilocarpine-induced SE model, original, uncropped blots are shown in Supplementary Fig. 1; **B**, Kainic acid-induced SE model, original, uncropped blots are shown in Supplementary Fig. 2; **C**, PTZ-induced seizures, original, uncropped blots are shown in Supplementary Fig. 3; **D**, Kindling model, original, uncropped blots are shown in Supplementary Fig. 4; upper panels, reverse-transcription PCR results; lower panels, quantitative real-time PCR results. *VDR* mRNA levels were normalized to those of *Gapdh*. *n* = 3 for each timepoint. (**E**) Immunohistochemical staining of VDR protein in DG, CA3 and CA1 of hippocampus, followed by fluorescent Nissl staining. Hoechst acts a counterstaining marker for the nuclei. DG, dentate gyrus; CA1, Cornu Ammonis 1; CA3, Cornu Ammonis 3. Scale bar, 50 µm. (**F**) Western blot analysis of VDR expression at different timepoints after pilocarpine-induced seizure. Original, uncropped blots are shown in Supplementary Fig. 5; upper panels, western blot results; lower panels, quantitative western blot results. GAPDH immunoblotting verified equal loading, *n* = 3 for each timepoint. Each data point represents a result from an individual mouse. Mean ± SEM. values are presented as fold change relative to the unstimulated (kindling model) or vehicle-treated (Pilocarpine, Kainic acid and PTZ models) control value. * *P* < 0.05, ** *P* < 0.01 and *** *P*  *<* 0.001; one-way ANOVA with *post hoc* Dunnett’s test.

To detect *VDR* mRNA expression, the experimental starting points were defined as follows: the onset of SE in the pilocarpine-induced SE model, and the first occurrence of Stage 5 seizures in the KA-induced SE and PTZ-induced acute epilepsy models. Hippocampal tissues were dissected from the mice at the time points shown in Fig. 1A–C for the subsequent experiments.

### Spontaneous seizure monitoring

Eight weeks after successful SE induction with pilocarpine, the mice were placed on transparent grids equipped with a camera for monitoring their activities. The mice were monitored daily from 09:30 to 21:30 (12 h/day) for 4 weeks. Timepoints and duration of Stage ≥ 4 seizures were recorded. Based on these records, we analysed the differences in the number of seizures between each week among control diet, Vit.D-deficiency diet and Vit.D-overdose diet group mice.

### Electrically induced kindling model

The electrode implantation and kindling procedures have been described previously.^25^ Briefly, twisted-wire bipolar electrodes were implanted into the right amygdala of adult animals following anaesthesia induction (bregma, 1.2 mm; lateral, 2.8 mm; and depth, 4.9 mm below dura) for electrical stimulation and recording. After the surgery recovery period of 1 week, we used a train of stimuli (60 Hz for 1 s) for amygdala kindling stimulation. Constant current pulse monophasic waveforms were generated using a JL-C4 V2a stimulator (Shanghai Jialong Educational Instrument Factory). A 60 µA initial intensity with incremental intensities was used to determine the threshold of evoked electrographic seizure (EST) in individual mice. EST current intensity was subsequently administered according to the standard amygdala kindling protocol.

The mice were electrically stimulated twice daily with a stimulation interval of >8 h for 15 days. A single electrical stimulation was performed 1 week later. The seizure stage and duration were recorded. Mice with three consecutive Stage ≥ 5 seizures during the 15-day stimulation procedure and Stage 4 or 5 seizures after the last stimulation were considered fully kindled.

To examine the *VDR* mRNA expression levels in the mouse brain after epilepsy, we performed the last single electrical stimulation to induce seizure activity. Mouse hippocampi were collected for RNA extraction at the indicated time points (Fig. 1 D).

### Immunofluorescence staining

The mice were sacrificed and perfused with 4% paraformaldehyde. Mouse brains were post-fixed at 4°C overnight. Coronal slices (30 µm thick) were prepared using a cryostat (Leica Microsystems, Germany). Sections were blocked with 10% normal donkey serum in phosphate-buffered saline with 0.3% Triton X-100 and then incubated with primary antibodies overnight, followed by washing and incubation with Alexa Fluor series (Invitrogen, USA) secondary antibodies for 2 h. The primary antibody used was a mouse anti-VDR antibody (1/500; SC-13133, Santa Cruz Biotechnology). For Nissl staining, brain sections were incubated with Neuro Trace 500/525 fluorescent Nissl stain (N21480, Thermo Fisher Scientific) for 15 min.

### Western blotting

Tissues were homogenized by RIPA buffer containing protease inhibitors, followed by centrifugation to remove debris at 12 000 rpm for 20 min at 4°C. Protein concentrations were measured using the bicinchoninic acid protein assay kit and denatured by heating at 100°C for 10 min. All protein samples were separated via 10% SDS‒polyacrylamide gel electrophoresis (Bio-Rad) and blotted onto PVDF membranes (Millipore). The membranes were blocked with 5% nonfat milk in 0.05% Tween 20 at room temperature for 1 h and probed with mouse anti-VDR antibody (1/1000; SC-13133, Santa Cruz Biotechnology) and GAPDH (1/10000; KC-5G4, Aksomics). For secondary antibodies, we used HRP-conjugated goat anti-mouse immunoglobulin G (IgG) secondary antibodies (KC-MM-035; Kangchen).

### Timm staining

Mice were transcardially perfused with an ice-cold solution containing 1.2% H_2_S (w/v) and 1% NaH_2_PO_4_ (w/v) in distilled water for 5 min, followed by 4% paraformaldehyde (PFA). The staining solution was prepared by mixing 50% gum arabic (w/v), 5.67% hydroquinone (w/v), 26% citric acid-sodium citrate buffer (26% citric acid + 24% sodium citrate, w/v) and 17% silver nitrate (w/v; all from Sigma–Aldrich) in a 12/6/2/1 volume ratio. Horizontal brain sections (30 μm) were incubated with the staining solution for 45 min at room temperature. Timm-stained hippocampal dentate gyrus images were acquired using a Thermo Fisher EVOS FL AUTO 2 microscope. Quantitative analysis was performed with Image Pro-Plus 6.0 (Media Cybernetics) by calculating the Timm index.

Timm Index = Total Timm granule area/Dentate gyrus linear length

### Bioinformatics analysis

To identify overlapping targets between Vit.D and epilepsy, we performed an intersection analysis by cross-referencing previously reported Vit.D-related genes,^26^ with epilepsy-associated genes from the Seizure-Associated Genes Across Species (SAGAS) database (https://onedrive.live.com/:x:/g/personal/5e8dec6430c197c9/UQDJl8EwZOyNIIBeigAAAAAAAHCLY-UZxa0Vc4U?rtime=qYL8PUZw3kg&redeem=aHR0cHM6Ly8xZHJ2Lm1zL3gvcyFBc21Yd1RCazdJMWVnUXB3aTJQbEdjV3RGWE9GP2U9RDhneTBV)^27^ and subsequently visualized the conserved gene sets using Venn diagrams. The overlapped genes were subjected to the Gene Ontology (GO) function and Kyoto Encyclopaedia of Genes and Genomes (KEGG) pathway enrichment analyses using an online platform (www.bioinformatics.com.cn). The analysis was limited to *Homo sapiens*, with significance set at *P* ≤ 0.05. Subsequently, the overlapped genes were imported into the STRING database to construct a protein–protein interaction (PPI) network and the network topology was analysed using Cytoscape 3.10.3 software. Furthermore, Cytoscape software was applied for hub target identification through the cytoHubba plugin's Maximal Clique Centrality (MCC) algorithm.

### RNA extraction and reverse transcription polymerase chain reaction

Fresh brain tissue samples were collected from C57BL/6 epileptic mice at different timepoints, as indicated. The brain tissue samples were carefully collected on ice transferred into the RNase-free Eppendorf tubes, and temporarily stored at −80°C in a freezer. After all the brain tissue samples from each time point were collected, RNA was extracted and cDNA was synthesized using a reverse transcription kit (Invitrogen). The primers were designed according to open source Internet-based *in situ* gene expression data and were synthesized by Invitrogen.^28^ The primers used in the experiments as follows. *Gapdh*, Forward primer, CCCCAATGTATCCGTTGTG; Reverse primer, CTCAGTGTAGCCCAGGATGC; *VDR*, Forward primer, ACCGCCTATCCACACACTG; Reverse primer: TTGCCGAACACCTCTAGCAC. Polymerase chain reaction (PCR) was performed according to standard procedures described previously.^25,29^ Thereafter, PCR products were electrophoresed on the 2% agarose gel at a constant current of 150 mA and imaged and analysed using a Tanon-2500 gel imaging system.

### Real-time PCR

Real-time PCR was performed using the SYBR Green I Premix Ex Taq Kit (TaKaRa Bio) according to the manufacturer’s instructions. The reaction solution was prepared using reagents provided in the SYBR Green I Premix Ex Taq kit. PCR was performed using the ABI Prism 7000 system.

### Statistical analysis

**Figure 2:**
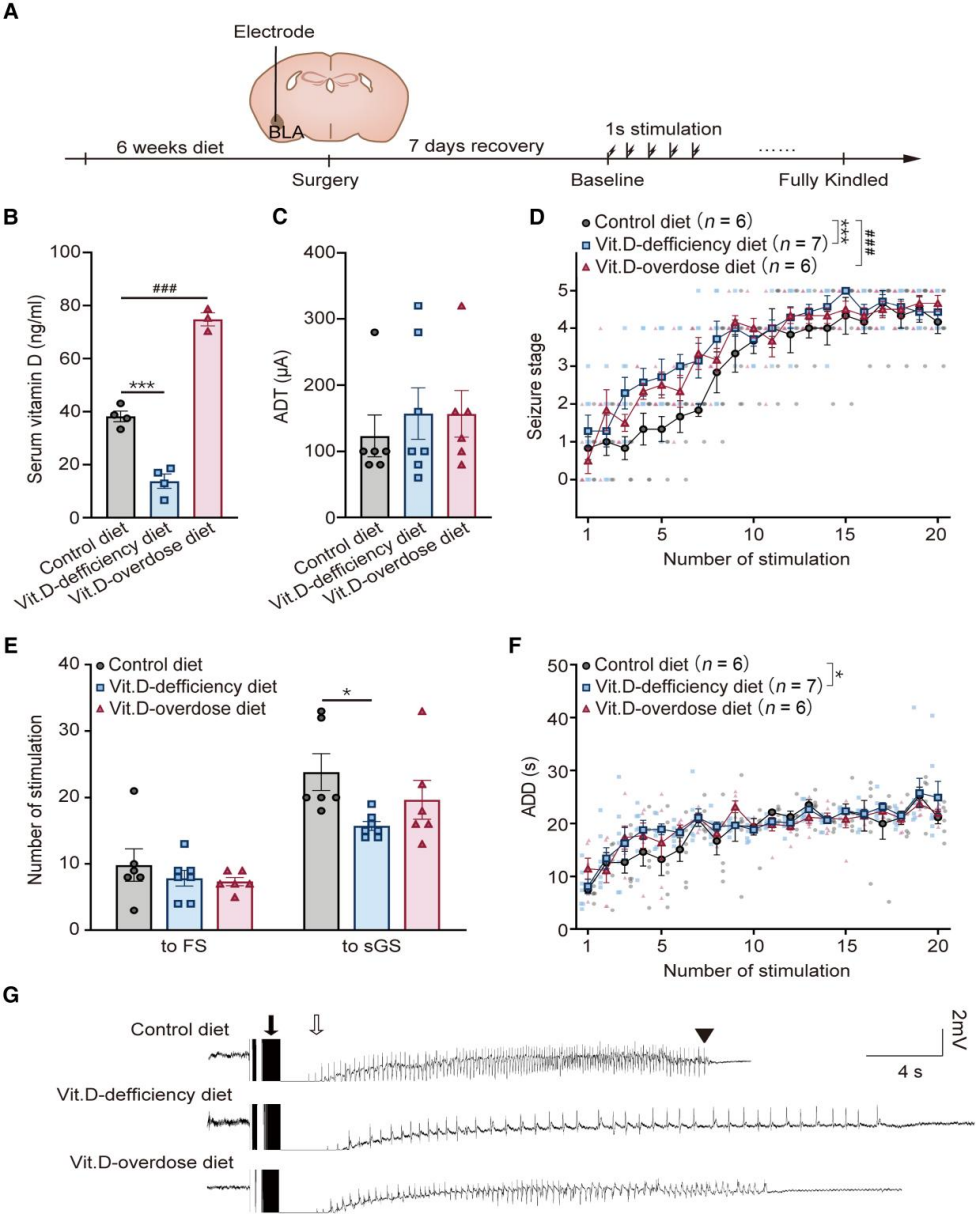
**Effects of Vit.D on kindling development of mouse models.** (**A**) Schematic of the protocol. (**B**) Serum 25-hydroxyvitamin **D** [25(OH)D] concentrations in control diet, Vit.D-deficiency diet and Vit.D-overdose diet group mice (*n* = 3 for control diet, *n* = 4 for Vit.D-deficiency diet and *n* = 3 for Vit.D-overdose diet). (**C**) Quantitative analysis of electrographic seizure threshold (EST) for control diet, Vit.D-deficiency diet and Vit.D-overdose diet groups. (**D–F**) Kindling development for control diet, Vit.D-deficiency diet and Vit.D-overdose diet group mice (*n* = 6 for control diet, *n* = 7 for Vit.D-deficiency diet and *n* = 6 for Vit.D-overdose diet). Grey, blue and red dots represent individual mice. **D**, Stimulation numbers required to reach equivalent seizure class in kindling development in the three models; a two-way ANOVA was undertaken with number of stimulation and diet as independent variables and showed that there was a significant main effect of number of stimulation [*F*(19 320) = 40.56, *P* < 0.0001 and diet[*F*(2320) = 17.13, *P* < 0.0001] with no significant interaction between the two terms [*F*(38 320) = 0.9231, *P* = 0.6033]; *post hoc* Bonferroni’s method: control diet *versus* Vit.D-deficiency diet: *P* ＜ 0.0001; control diet *versus* Vit.D-overdose diet: *P* = 0.0002. **E**, Number of stimulations required to reach the FS and sGS state in the three groups. **F**, The effect of Vit.D on the duration of after discharge duration (ADD) in epileptic seizures; a two-way ANOVA was undertaken with number of stimulation and diet as independent variables and showed that there was a significant main effect of number of stimulation [*F*(19 320) = 15.89, *P* < 0.0001] and diet [*F*(2320) = 3.027, *P* = 0.0498] with no significant interaction between the two terms [*F*(38 320) = 0.7452, *P* = 0.8642]; *post hoc* Bonferroni’s method: control diet *versus* Vit.D-deficiency diet: *P* = 0.0467; control diet *versus* Vit.D-overdose diet: *P* = 0.3431. (**G**) Typical EEG recordings at the first Stage-5 seizure. Filled arrow, application of electrical stimulation (truncated stimulus artefact); open arrow, *post*-stimulation refractory period; arrowheads, termination point of electrographic seizure. Each point represents a single animal, control diet *versus* Vit.D-deficiency diet, * *P* < 0.05, ** *P* < 0.01 and *** *P* < 0.001; control diet *versus* Vit.D-overdose diet, ^#^  *P* < 0.05, ^##^  *P* < 0.01 and ^###^  *P* < 0.001; the data represent the mean ± SEM; one-way ANOVA with *post hoc* Dunnett’s test in (**B**)–(**C**); two-way ANOVA with *post hoc* Bonferroni’s test in (**D**)–(**F**).

**Figure 3:**
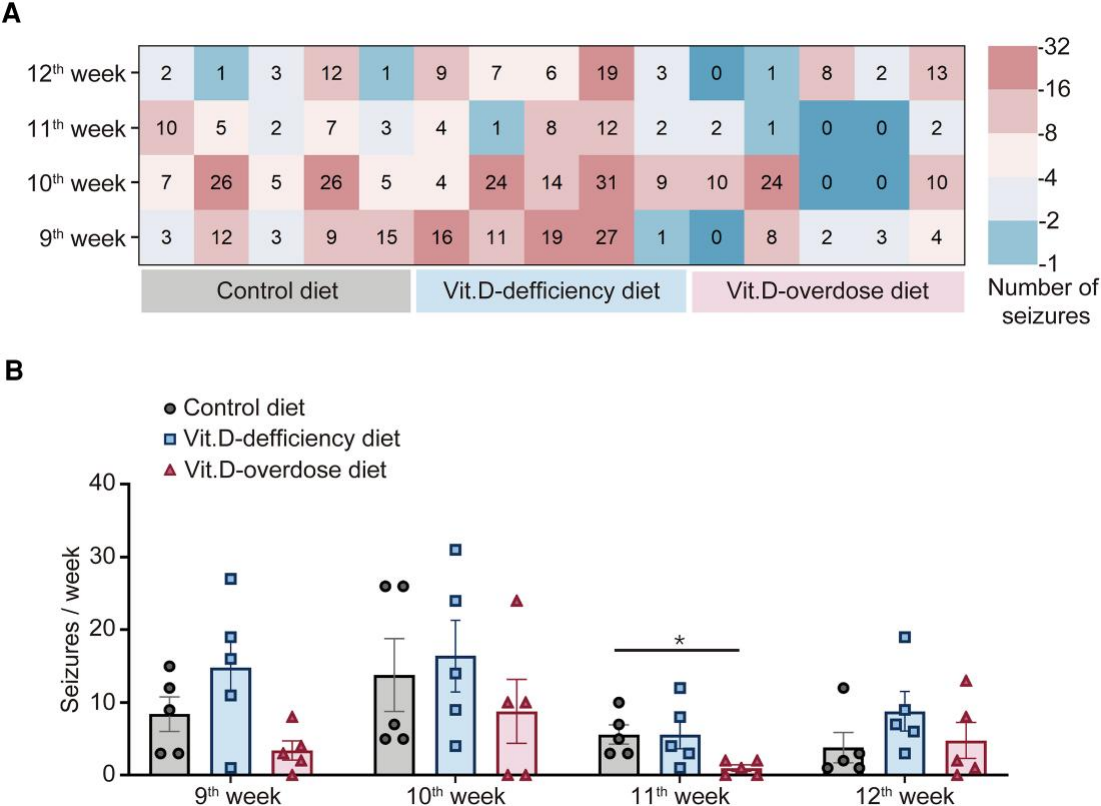
**Vit.D affects the occurrence of spontaneous seizures after pilocarpine-induced SE.** (**A**) Heatmap of spontaneous seizure events showing cumulative convulsive seizure onset frequency *per* week for individual mice from control diet, Vit.D-deficiency diet and Vit.D-overdose diet groups. (**B**) Statistical analysis of seizure frequency. *n* = 5 for each group. Each point represents a single animal, * *P* < 0.05; the data represent the mean ± SEM, One-way ANOVA on Ranks with Dunn's *post hoc* test corrected by the Bonferroni method.

**Figure 4:**
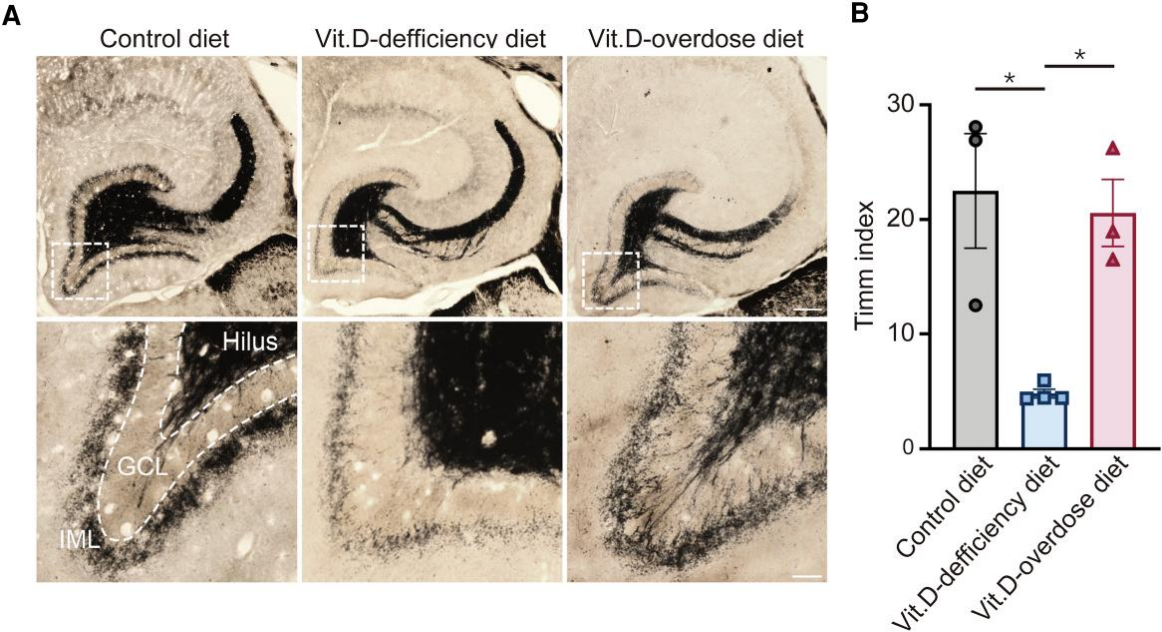
**Vit.D-deficiency decreases mossy fibre sprouting.** (**A**) Representative images of Timm staining for mossy fibre sprouting in mouse hippocampus 3 months after pilocarpine-induced SE from control diet, Vit.D-deficiency diet and Vit.D-overdose diet group mice. The regions indicated by dotted lines in the upper panels are shown at higher magnification below. Scale bars, 500 µm (top row) and 50 µm (bottom row). (**B**) Statistical analysis of mossy fibre sprouting results as revealed by Timm index. *n* = 3–4 for each group. Each point represents a single animal, * *P* < 0.05; the data represent the mean ± SEM, one-way ANOVA with *post hoc* Bonferroni’s test.

The investigators were blinded to the treatment groups for all the statistical analyses. Timm index data analysis was performed using Image-Pro Plus 6.0 software. SPSS software (version 26.0) was used for the analysis of reverse transcription (RT)-PCR and real-time PCR data, kindling data, spontaneous seizure frequency and Timm index. Prism 8.0 software was used to produce the statistical graphs. Data are presented as mean ± standard error of mean (SEM). The significance of the differences between each group was tested and analysed using one-way ANOVA with *post hoc* Dunnett’s test in Fig. 1; one-way ANOVA with *post hoc* Dunnett’s test and two-way ANOVA with *post hoc* Bonferroni test in Fig. 2; One-way ANOVA on Ranks with Dunn's *post hoc* test corrected by the Bonferroni method in Fig. 3 due to unequal variances; one-way ANOVA with *post hoc* Bonferroni test in Fig. 4. Statistical significance was defined as *P* < 0.05.

## Results

### Seizure activity upregulates VDR expression in the mouse hippocampus

First, we used the pilocarpine model, which is the classic animal model of limbic epilepsy, and tested the mRNA levels of *VDRs* at different timepoints following seizure occurrence (Fig. 1). RT-PCR results revealed that *VDR* mRNA expression was markedly upregulated soon after 6 h and peaked at 24 h following seizure activity, with a nearly 13.5-fold increase compared to that of baseline (6 h, *P* = 0.0419; 12 h, *P* = 0.0102; 24 h, *P* = 0.0021). Thereafter, the *VDR* mRNA level began to gradually decrease and was restored to the normal baseline level on the third day following limbic seizure activity. Real-time PCR confirmed these changes in animal models (Fig. 1A). To exclude the possible direct effects of pilocarpine on *VDR* mRNA expression, we employed another widely used epilepsy model—KA-induced seizure activity model in mice. As observed in the pilocarpine model, *VDR* mRNA expression was also elevated by epileptic seizures at 6 h, which was 9.5-fold that of the control (*P* = 0.0037), as demonstrated by both RT-PCR and real-time PCR results (Fig. 1B). These results indicate that *VDR* expression is upregulated after limbic seizure activity.

To determine whether the upregulation of *VDR* gene expression is a general phenomenon in limbic epilepsy, we subsequently used the PTZ-induced seizure model to confirm the abovementioned findings. Consistently, *VDR* expression levels peaked 6 h after epileptic seizures, reaching about 2.7-fold that of the control group (*P* = 0.0004). However, the expression level of *VDR* mRNA began to decrease after 12 h and was restored to the baseline levels at 24 h (Fig. 1C), possibly owing to the mild seizure activity with a short duration of Stage 5 seizures.^30^

To exclude the possible pharmacological roles of the above chemical agents in regulating *VDR* gene expression within the mouse brain, we subsequently observed the changes in the *VDR* transcript levels after single electrical stimulation-induced seizure. Notably, the *VDR* mRNA expression in the hippocampus increased after the single electrical stimulation-induced seizure, peaking at 6 h, and was approximately 3.5-fold that of the unstimulated group (*P* = 0.0088). Consistent with the results obtained from the PTZ model, the *VDR* mRNA levels started to decrease 12 h after kindled seizure activity, as revealed by the RT-PCR and real-time PCR (Fig. 1D).

Next, to investigate the spatiotemporal dynamics of the VDR in epileptogenic hippocampal networks, we performed immunofluorescence (IF) staining of the brain sections. VDR-expressing neurons, including those containing Nissl bodies, were localized in the hippocampus. Following SE induction, VDR immunoreactivity was markedly increased across hippocampal subregions, with the most pronounced signals in limbic epilepsy-associated subfields (Fig. 1E). Consistently, western blot analysis revealed a rapid elevation of VDR protein levels after pilocarpine administration, peaking at 6 h post-injection before declining, with the 6 h time point showing a statistically significant increase compared with controls (*P* = 0.0197; Fig. 1F). Together, these results demonstrate that seizure activity triggers a transient but robust upregulation of VDR expression in the hippocampus, suggesting a potential functional role in epileptogenesis.

### Aberrant Vit.D dietary levels could affect kindling development

The advantage of the kindling model lies in its ability to systematically quantify and track the seizure activity in epileptogenesis through standardized behavioural assessments and electroencephalographic recording.^1^ In contrast to other models, the electric kindling model is a chronic model of epilepsy that produces molecular and cellular alterations in the mouse brain induced by repeated initial electrical stimuli, resulting in the progressive intensification of seizures, culminating in generalized tonic-clonic seizures.^31^ We investigated the role of Vit.D in epileptogenesis using the mouse kindling model by manipulating systemic levels through either a Vit.D-deficiency or -overdose diet (Fig. 2A). To assess systemic Vit.D status in the dietary intervention groups, serum 25-hydroxyvitamin D [25(OH)D] levels were measured. Mice fed the control diet displayed mean serum concentration of 38.19 ± 2.01 ng ml⁻¹ (mean ± SEM). In comparison, those on the Vit.D deficiency diet exhibited a significant reduction, with levels falling to 13.79 ± 2.66 ng ml⁻¹ (mean ± SEM, *P* = 0.0001 *versus* control diet; Fig. 2B). Conversely, mice receiving the Vit.D-overdose diet exhibited elevated levels to 74.78 ± 2.45 ng ml⁻¹ (mean ± SEM, *P* < 0.0001 *veversus* control diet; Fig. 2B). These results confirmed the efficacy of the dietary manipulations in establishing distinct systemic Vit.D states (Fig. 2B). Our results revealed that the intake of different Vit.D contents had no effect on the electrographic seizure threshold of the mice [mean ± SEM, control diet, 123.3 ± 31.59 µA; Vit.D-deficiency diet, 157.1 ± 38.90 µA; Vit.D-overdose diet, 156.7 ± 35.18 µA; one-way ANOVA, *F*(2, 16) = 0.2838, *η*_p_^2^ = 0.034, *P* = 0.7567; *post hoc* Dunnett’s method, control diet *versus* Vit.D-deficiency diet, Cohen’s *d* = −0.37, 95% confidence interval (CI) (−155.5 to 87.88), *P* = 0.7274; control diet *versus* Vit.D-overdose diet, Cohen’s *d* = −0.41, 95%CI (−159.6 to 92.95), *P* = 0.7492; Fig. 2C], indicating that Vit.D intake could not alter the baseline sensitivity to kindling stimulation. A Vit.D-deficiency model was used to assess the role of Vit.D in epileptogenesis. Compared with control diet, Vit.D-deficiency diet significantly accelerated the progression of behavioural seizure class (*P* < 0.0001 *versus* control diet; Fig. 2D), reduced the number of electrical stimulations required to reach sGS State (mean ± SEM, control diet, 23.8 ± 2.8; Vit.D-deficiency diet, 15.7 ± 0.7; *P* = 0.0154 *versus* control diet; Fig. 2E), increased the prolongation of electro graphic seizure duration (ESD) (*P* = 0.0467 *versus* control diet; Fig. 2F). Typical EEG of ESD in the initial Stage-5 seizures was shown (Fig. 2G). These findings suggest that Vit.D-deficiency accelerates kindling-induced epileptogenesis compared to that in the control group.

Moreover, long-term excess Vit.D intake can be harmful.^32^ We examined the effects of excess Vit.D intake on epileptogenesis. An increased number of electrical stimulations were required to evoke stereotypical behavioural seizures in the Vit.D-overdose diet group mice (*P* = 0.0002 *versus* control diet; Fig. 2D), suggesting that consuming excessive amounts of Vit.D daily affects seizure progression induced by stimulation.

### Vit.D supplement alleviates pilocarpine-induced chronic spontaneous seizures

The tendency of pilocarpine-induced SE to lead to spontaneous seizures makes it a prevalent model for research into epileptogenic mechanism.^33^ To investigate the effect of Vit.D on pilocarpine-induced spontaneous seizures, we adjusted systemic Vit.D levels as previously described following the induction of SE. Subsequently, all mice underwent continuous video monitoring for four weeks, commencing two months post-SE. The weekly seizure frequency for each mouse is shown in Fig. 3A. There is no significant difference in spontaneous seizure frequency among the control diet, Vit-D-deficiency diet and Vit.D-overdose diet mice during the 9th week, 10th week or 12th week (ANOVA on Ranks, 9th week, *P* = 0.0975; 10th week, *P* = 0.5774; 12th week, *P* = 0.2615). Mice in the Vit.D-overdose diet group demonstrated a significantly lower frequency of spontaneous seizures compared with the control group during the 11th week (mean ± SEM, control diet, 5.6 ± 1.3; Vit.D-deficiency diet, 5.6 ± 2.0; Vit.D-overdose diet, 1.0 ± 0.4; ANOVA on Ranks, *P* = 0.0112; *post hoc* Dunn’s method, control diet *versus* Vit.D-deficiency diet, *P* > 0.9999; control diet *versus* Vit.D-overdose diet, *P* = 0.0346; Vit.D-deficiency diet *versus* Vit.D-overdose diet, *P* = 0.0684; Fig. 3B). These results indicated that the supplementing Vit.D suppresses seizure frequency in epileptic mice.

### Vit.D is required for the development of mossy fibre sprouting in the pilocarpine model

Mossy fibre sprouting, a hallmark chronic neuropathological manifestation in TLE, is characterized by aberrant axonal sprouting of hippocampal mossy fibres that infiltrate the inner molecular layer of the DG. It typically occurs several weeks following recurrent epileptic episodes and exhibits strong correlations with both the initiation and progression of epileptogenesis.^34^ These findings suggest that Vit.D is closely associated with seizure-induced long-term alteration of neural circuitry. To investigate whether the changes in the Vit.D levels affect the mossy fibre sprouting in the limbic epileptic brain, we performed Timm staining to observe the alterations of mossy fibre sprouting in the hippocampus of the mice experienced SE (Fig. 4A). We found that Vit.D-deficiency diet group mice showed no overt pathological mossy fibre sprouting. The Timm index was significantly lower in the Vit.D-deficiency diet group mice, whereas the Vit.D-overdose diet group mice displayed no significant difference in Timm index relative to the control diet groups (mean ± SEM, control diet, 22.50 ± 5.01; Vit.D-deficiency diet, 4.82 ± 0.38; Vit.D-overdose diet, 20.56 ± 2.92; one-way ANOVA, *F*(2, 7) = 11.63, *η*_p_^2^ = 0.77, *P* = 0.0060; *post hoc* Bonferroni’s method, control diet *versus* Vit.D-deficiency diet, Cohen’s *d* = 2.87, 95% CI (4.805–17.56), *P* = 0.0108; control diet *versus* Vit.D-overdose diet, Cohen’s *d* = 0.27, 95%CI (−11.82 to 15.71), *P* > 0.9999; Vit.D-deficiency diet *versus* Vit.D-overdose diet, Cohen’s *d* = −4.35, 95% CI(−28.62–2.861), *P* = 0.0196; Fig. 4B). These results indicate that Vit.D is critical for mossy fibre sprouting in the dentate gyrus of the mouse hippocampus.

### Identification of Vit.D-related genes, pathways and cellular functions during epileptogenesis

**Figure 5:**
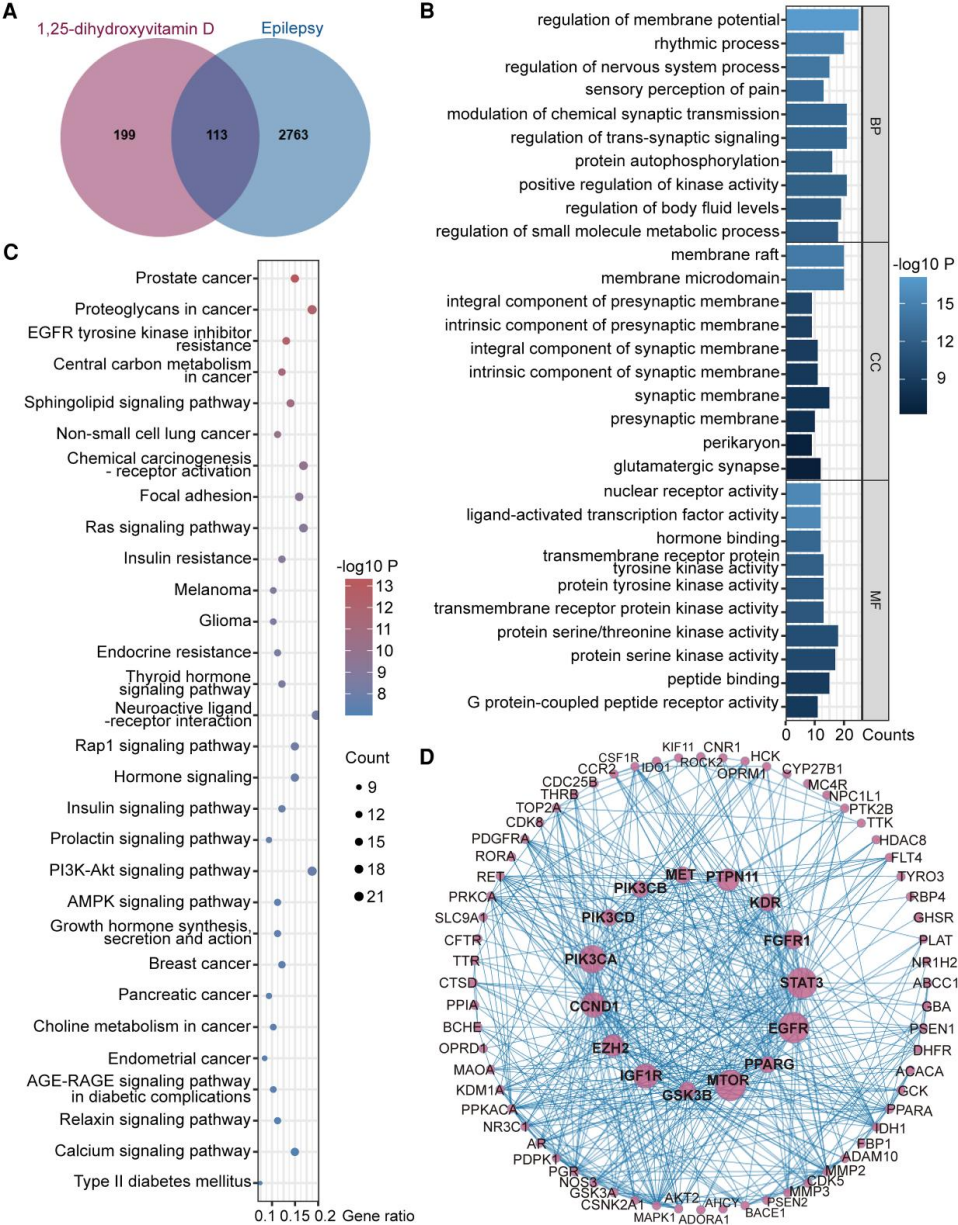
**Bioinformatic analysis of the role of Vit.D during limbic epileptogenesis.** (**A**) Venn diagram depicting the intersection targets between Vit.D-related targets and the epilepsy-associated targets. (**B**) GO pathway analysis of the potential targets of Vit.D in the treatment of epilepsy. (**C**) A bubble chart shows the impact of KEGG pathway enrichment analysis of potential Vit.D targets on limbic epileptogenesis. (**D**) A PPI network map of core intervention targets.

We acquired the differentially expressed genes (DEGs) in epilepsy set from the SAGAS database,^26^ which included 2876 genes, and 312 Vit.D-related genes (VRGs) were collected from various databases.^27^ Cross-referencing the VRGs set with the DEGs allowed us to identify 113 VRGs including VDR. These VRGs were visually represented in a Venn diagram (Fig. 5A). The 113 differential genes were annotated into cellular composition, main biological processes and molecular function in the GO function classification analysis (Fig. 5B). The results from the GO analysis revealed that these genes are predominantly localized to the synapse, playing crucial roles in the regulation of synaptic transmission. Consequently, the top 30 results from the KEGG analysis were visualized using the Weishengxin data analysis platform, resulting in a bubble map for visualization (Fig. 5C). The overlapping genes of Vit.D and epilepsy were input into the STRING database, generating a PPI network map. Key intervention targets primarily included *STAT3, EGFR, MTOR, PIK3CA, IGF1R, CCND1, PTPN11, EZH2, FGFR1, KDR, GSK3B, MET, PPARG, PIK3CB, PIK3CD* (Fig. 5D).

## Discussion

In this work, we present evidence that Vit.D is critical for inhibiting limbic epileptogenesis and report the predicted mechanism. First, seizure activity increases the expression of VDR in the brain, especially in hippocampal dentate gyrus, which is a critical area for limbic seizure initiation and E/I balance, implying that Vit.D signalling is dynamically regulated by epileptic activity, positioning a compensatory brain response to seizure-induced stress. This aligns with the ‘neuroprotective barrier’ hypothesis and reactive plasticity theories, wherein the brain attempts to restore ionic and synaptic balance post-injury. Second, the aberrant serum Vit.D levels affected limbic epileptogenesis, may be closely related to Vit.D-relevant changes in the brain microenvironment. Third, the deficiency of Vit.D affected mossy fibre sprouting in the dentate gyrus region of the mouse hippocampus, which may connect systemic Vit.D status to anatomical remodelling of seizure circuits. Fourth, bioinformatics analysis suggests that Vit.D may affect epileptogenesis by PI3K-AKT-mTOR signalling pathway. Together, these results indicate that aberrant Vit.D dietary levels could affect epileptogenesis, enhancing our understanding of the mechanism and pathological changes of limbic epilepsy.

Epilepsy is a chronic neurological disorder characterized by recurrent spontaneous seizures resulting from aberrant neuronal hyperexcitability.^1^ Hence, therapeutic interventions based on the pathogenesis of epilepsy, which could reduce the frequency and severity of seizures, might be helpful. As a steroid hormone, Vit.D has a variety of functions in the central nervous system (CNS), which plays a regulatory role in neurogenesis,^35^ intracellular calcium homeostasis regulation^36^ and chronic inflammation.^37^ Its receptor, VDR, participates in a variety of physiological activities.^38-46^ The occurrence of epilepsy is closely associated with disturbances in calcium homeostasis,^47^ chronic inflammation^46^ and abnormalities in neurotrophic factors,^48^ which likely are associated with the role of Vit.D in epilepsy.

Seizure activity triggered by diverse stimuli initiates a signalling cascade that culminates in the expression of numerous genes. This process is closely associated with cytoprotection, regulation of neurotransmitter synthesis and metabolism and activation of the immune system.^49-51^ We observed that VDR is upregulated by seizure activity in four typical multiple limbic epilepsy models. Although, the extent of VDR upregulation varies in different experimental models, reflecting its potential relationship with the differences in seizure severity, neuronal injury and mechanisms of epileptogenesis in distinct animal models. Both pilocarpine- and KA-induced models, which reliably induce SE, are simultaneously accompanied by more severe and widespread neuronal damage and inflammation,^52^ which may trigger a more robust response in the form of VDR upregulation. In the PTZ-induced model, PTZ acts mainly as a γ-aminobutyric acid (GABA) receptor antagonist, disrupting inhibitory neurotransmission in the brain.^52^ This leads to a relatively more restricted pattern of neuronal hyperexcitability and is milder than the seizures induced in other models. This may explain the low VDR upregulation in the PTZ-induced model.

In pilocarpine-treated rodents, the upregulated VDR was most prominently observed in the hippocampus, a region of critical importance for limbic epileptogenesis (Fig. 1).^53^This finding suggests that an increased transport of Vit.D may be necessary for the brain following seizure activity.

The kindling model is a classic animal model with repeatedly induced seizures resulting in increasing seizure duration and enhanced behavioural seizures, which is usually used for the study of epilepsy pathogenesis and preclinical anti-epilepsy drug evaluation.^54^ Using this model, we determined the effects of Vit.D on the kindling progress of limbic epilepsy. We observed that Vit.D-deficiency diet accelerated kindling-induced epileptogenesis (Fig. 2), consistent with clinical observation Meanwhile, Vit.D-overdose diet treatment also affected seizure progression induced by stimulation in epileptic seizure behaviour, which may associated with Vit.D-related hypercalcemia.^55^ However, in our model, we do not observe Vit.D could affect the seizure threshold, which is somewhat inconsistent with previous studies.^56^ This difference may be related to the difference in the route of administration and stimulation method.

The pilocarpine-induced model of SRSs is widely utilized as a representation of TLE, closely resembling TLE in humans.^57^ We analysed the behavioural associations of seizures occurring during SRSs after consuming different Vit.D dose and found that the mice in the Vit.D-overdose diet group showed fewer spontaneous seizures (Fig. 3), consistent with findings from previous reports in PTZ-treated animals. In PTZ-treated mice, the high dose of Vit.D demonstrated significant anticonvulsant effects,^58^ confirming the accuracy of our findings. Therefore, supplementing Vit.D may suppress the progression of epilepsy.

Mossy fibre sprouting is a chronic pathological feature of limbic epilepsy characterized by the abnormal growth of hippocampal mossy fibres into the inner molecular layer of the dentate gyrus.^59^ Such growth typically occurs several weeks after repeated seizure activity and is regarded as a structural correlate of recurrent excitatory circuitry that contributes to chronic hyperexcitability.^57^ Notably, we report for the first time that Vit.D-deficiency decreases mossy fibre sprouting, evidenced by decreased Timm staining intensity in hippocampal brain slices (Fig. 4). Previous work has demonstrated that mossy fibre sprouting involves abnormal axonal regeneration of dentate granule cells and synaptic reorganization—a process dependent on neurotrophic factors such as BDNF,^60^ NGF^61^ and NT-3.^62^ Given that Vit.D has been shown to upregulate the levels of these molecules,^63,64^ its deficiency could reduce aberrant mossy fibre sprouting by limiting them.

Additionally, our results showed that Vit.D deficiency reduced mossy fibre sprouting without altering seizure frequency, whereas Vit.D supplementation suppressed seizures without affecting mossy fibre sprouting (Figs 3 and 4). This suggests that Vit.D plays distinct roles in spontaneous seizures and mossy fibre sprouting. Although mossy fibre sprouting is traditionally viewed as a pro-epileptogenic process,^65^ some recent reports suggest that mossy fibre sprouting, a structural neuronal plasticity,^66^ represents a homeostatic mechanism that maintains excitatory synaptic input to granule cells in response to synapse loss after an epileptogenic injury, but it does not give rise to epileptic state.^60,67-69^ This process involves molecules that are associated with structural neuronal plasticity.^70^ As a positive regulator of neuronal structural plasticity,^46^ Vit.D interacts with perineuronal nets^71^; thereby, its deficiency may weaken both synaptic connectivity and perineuronal net integrity. Our findings are the first to present Vit.D as a novel molecule whereby its deficiency may reduce seizure-induced mossy fibre sprouting. This could enhance our understanding of the pathological changes associated with limbic epilepsy and suggests the complex role of Vit.D in shaping the epileptic brain.

Our bioinformatic analysis suggested that Vit.D may affect epileptogenesis through sustaining synaptic stability (Fig. 5B), with the PI3K-AKT-mTOR signalling pathway identified as a critical hub (Fig. 5C and D). This signalling pathway is well-established to regulate synaptogenesis,^72^ neuronal survival^73^ and excitability regulation,^74^ and its dysregulation is associated with seizure-related neuroinflammation^75^ and synaptic remodelling.^76^ Notably, experimental evidence suggests that targeted inhibition of this pathway can attenuate seizure severity and suppresses epileptogenesis.^77^ These findings may suggest that Vit.D affect synaptic function via the PI3K-AKT-mTOR signalling pathway during epileptogenesis, suggesting a potential therapeutic mechanism for Vit.D supplementation in epilepsy.

Although our murine models captured variations in the epileptogenesis of limbic epilepsy, the inherent complexity of the advanced CNS in humans has led to significant interspecies differences in the neural networks between rodents and humans.^78^ Future research should prioritize the establishment of epilepsy induction models across phylogenetically diverse species e.g. rats,^79^ cynomolgus macaques^80^ and beagle dogs,^81^ to quantitatively assess the effect of Vit.D during epileptogenesis. Future studies should employ targeted manipulations of VDR to investigate this mechanism.

In conclusion, our findings indicate a significant increase in Vit.D receptor expression after seizure activity, aberrant dietary Vit.D levels affect kindling development and induce spontaneous mossy fibre sprouting. These findings help us to understand the novel mechanisms of limbic epileptogenesis and provided some inspirations for the clinical management of epilepsy. A balanced clinical approach involves monitoring and maintaining Vit.D levels within a defined physiological range, since both deficiency and inappropriate excess supplementation may be suboptimal. Within this framework, adequate Vit.D supplementation via dietary therapy is beneficial for Vit. D-deficient individuals with epilepsy.

## Conclusions

In summary, our findings indicated that limbic seizure activity increased the expression of VDR in the brain, and aberrant Vit.D dietary levels could affect epileptic pathogenesis. Our work links neuroscience and nutrition, underscoring the role of Vit.D in the epileptic brain. However, further study is necessary to address the underlying mechanisms and potential applications in clinical settings comprehensively.

## Supplementary Material

fcag049_Supplementary_Data

## Data Availability

The raw data that support the findings of this study are available from the corresponding authors upon reasonable request. The Differentially Expressed Genes (DEGs) in epilepsy of Fig. 5A was from SAGAS database. The source data can be found under the following which is also referenced in the data availability statement (https://onedrive.live.com/:x:/g/personal/5e8dec6430c197c9/UQDJl8EwZOyNIIBeigAAAAAAAHCLY-UZxa0Vc4U?rtime=qYL8PUZw3kg&redeem=aHR0cHM6Ly8xZHJ2Lm1zL3gvcyFBc21Yd1RCazdJMWVnUXB3aTJQbEdjV3RGWE9GP2U9RDhneTBV). The bioinformatics analyses in this study (specifically for Fig. 5) were performed using the online analysis tools (www.bioinformatics.com.cn) and Cytoscape software. The original datasets generated for this study are included in the article and/or Supplementary materials. Any additional inquiries should be directed to the corresponding author.
