# Supplementary material for: Vitamin D is involved in regulating limbic epileptogenesis
Source: Brain Commun. 2026 Feb 16;8(2):fcag049. doi: 10.1093/braincomms/fcag049 (PMC13056719; doi:10.1093/braincomms/fcag049)
Supplement: fcag049_Supplementary_Data [file fcag049_supplementary_data.pdf]

Pilocarpine-induced SE

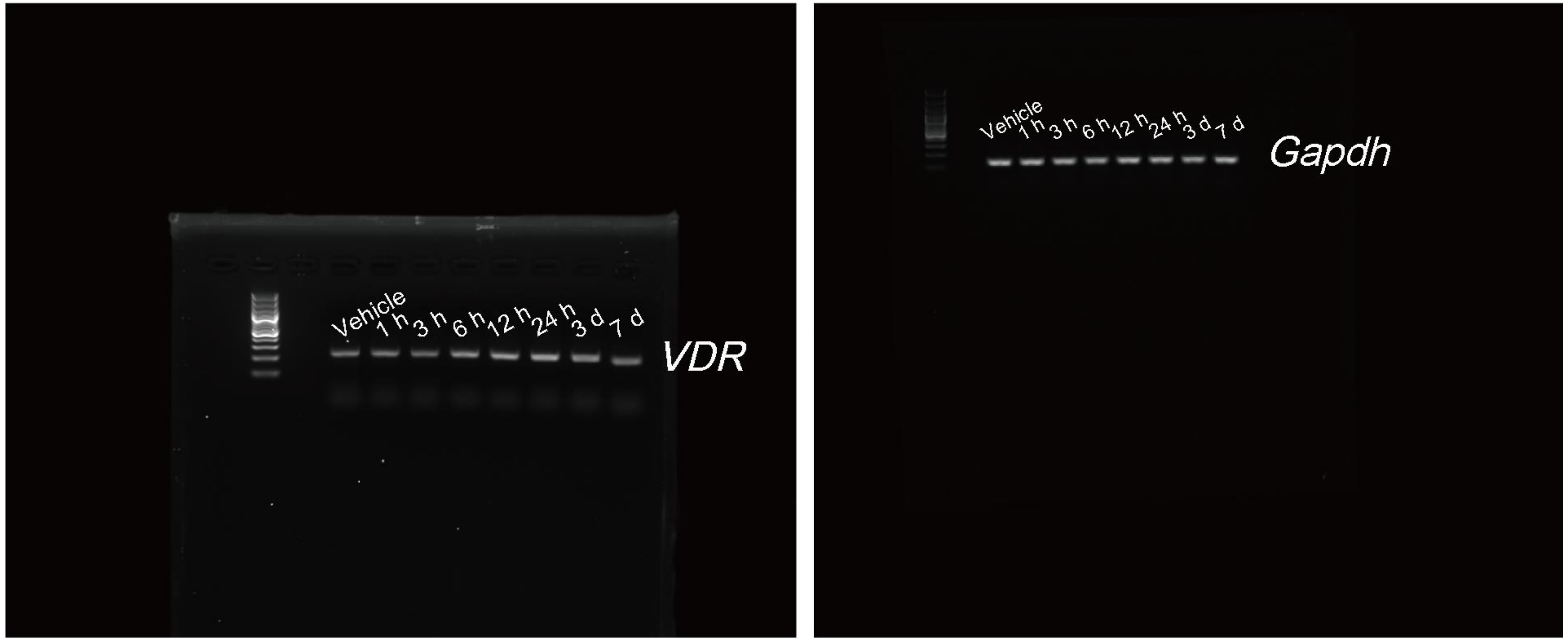

Supplementary Figure 1. Original, uncropped blots for Figure 1A

Kainic acid-induced SE

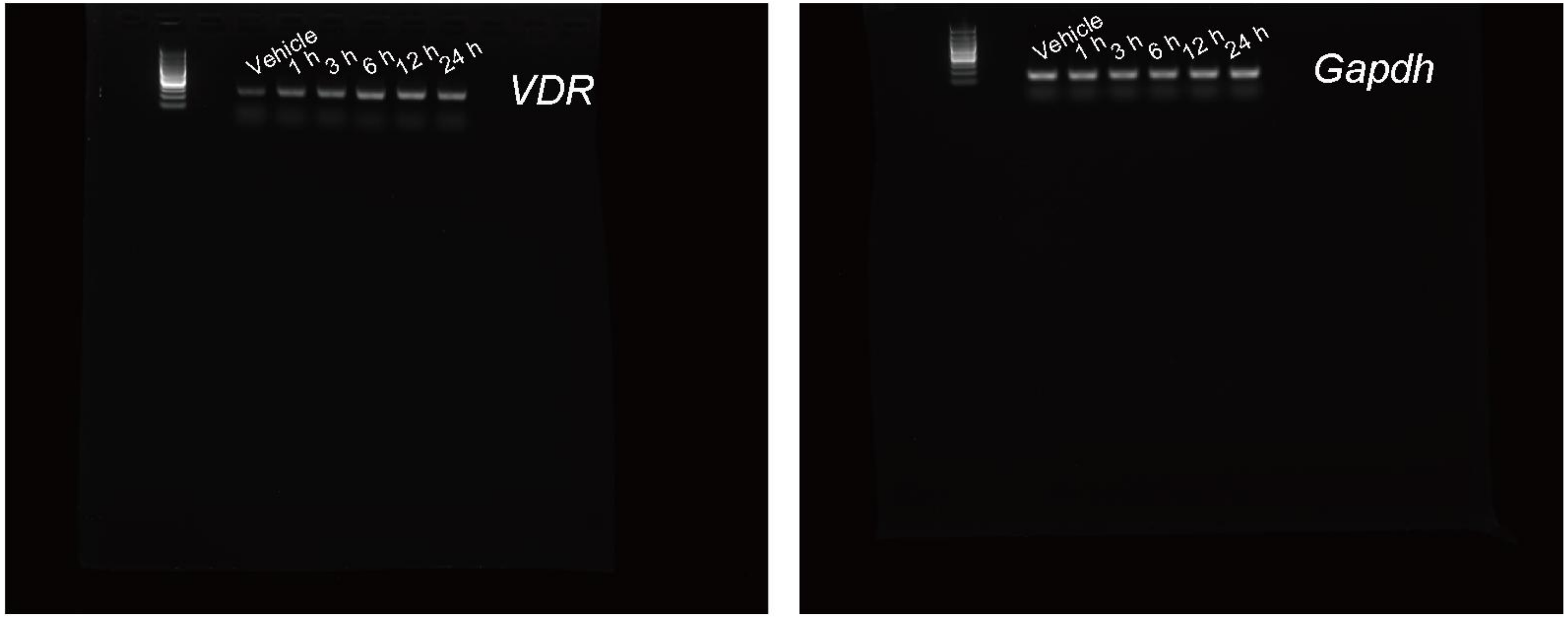

Supplementary Figure 2. Original, uncropped blots for Figure 1B

PTZ-induced seizure

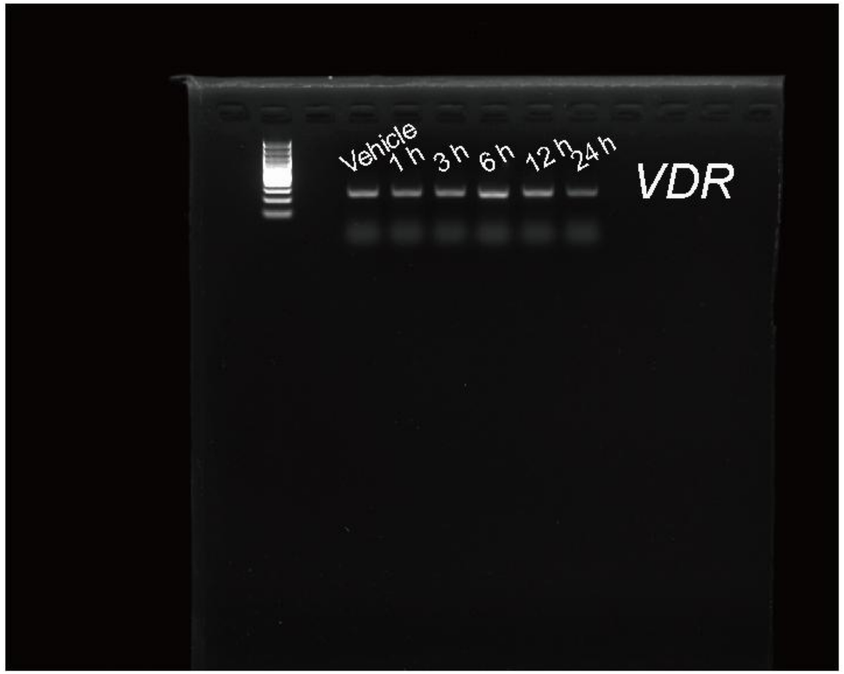

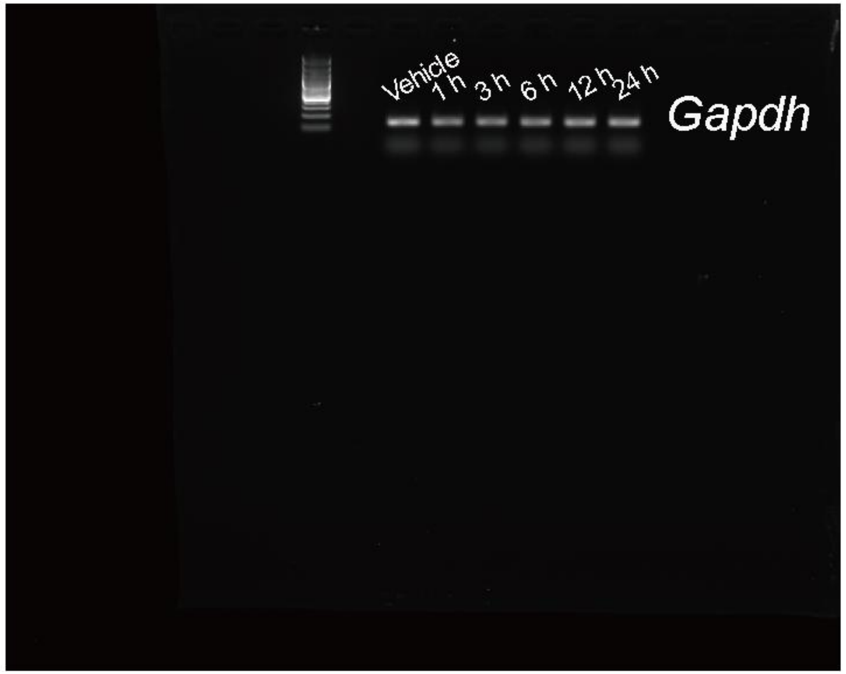

Supplementary Figure 3. Original, uncropped blots for Figure 1C

Kindling model

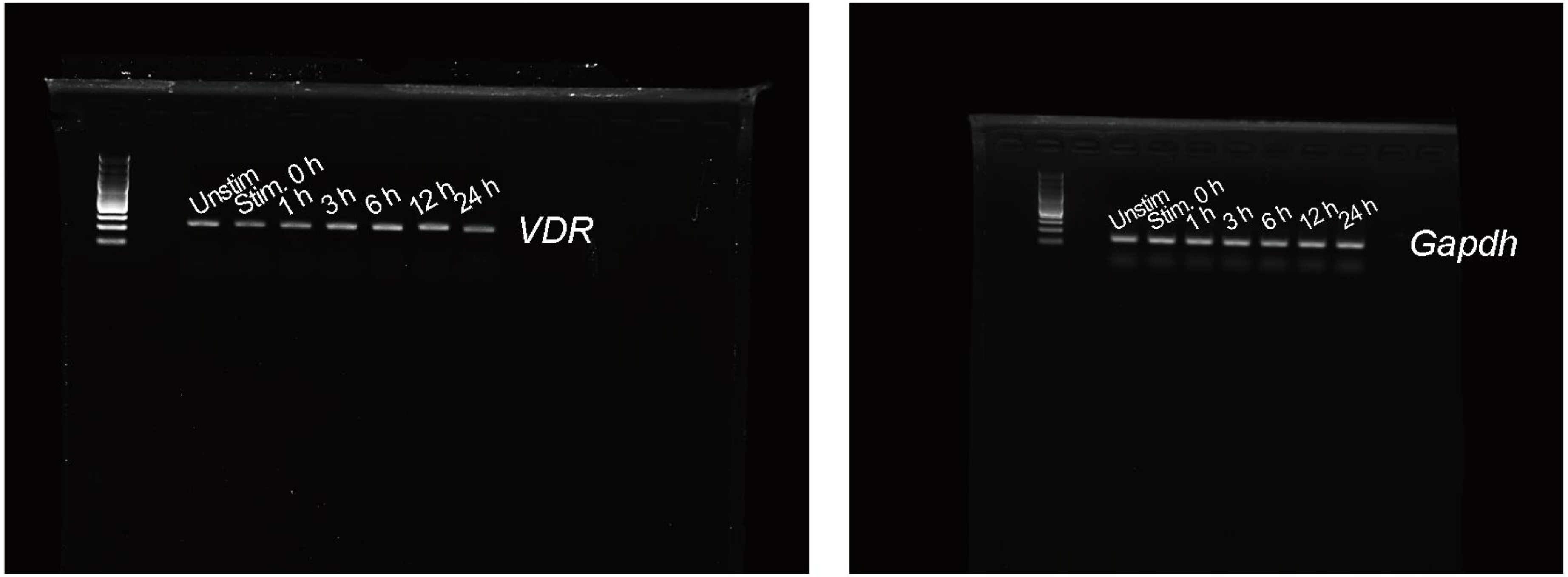

Supplementary Figure 4. Original, uncropped blots for Figure 1D

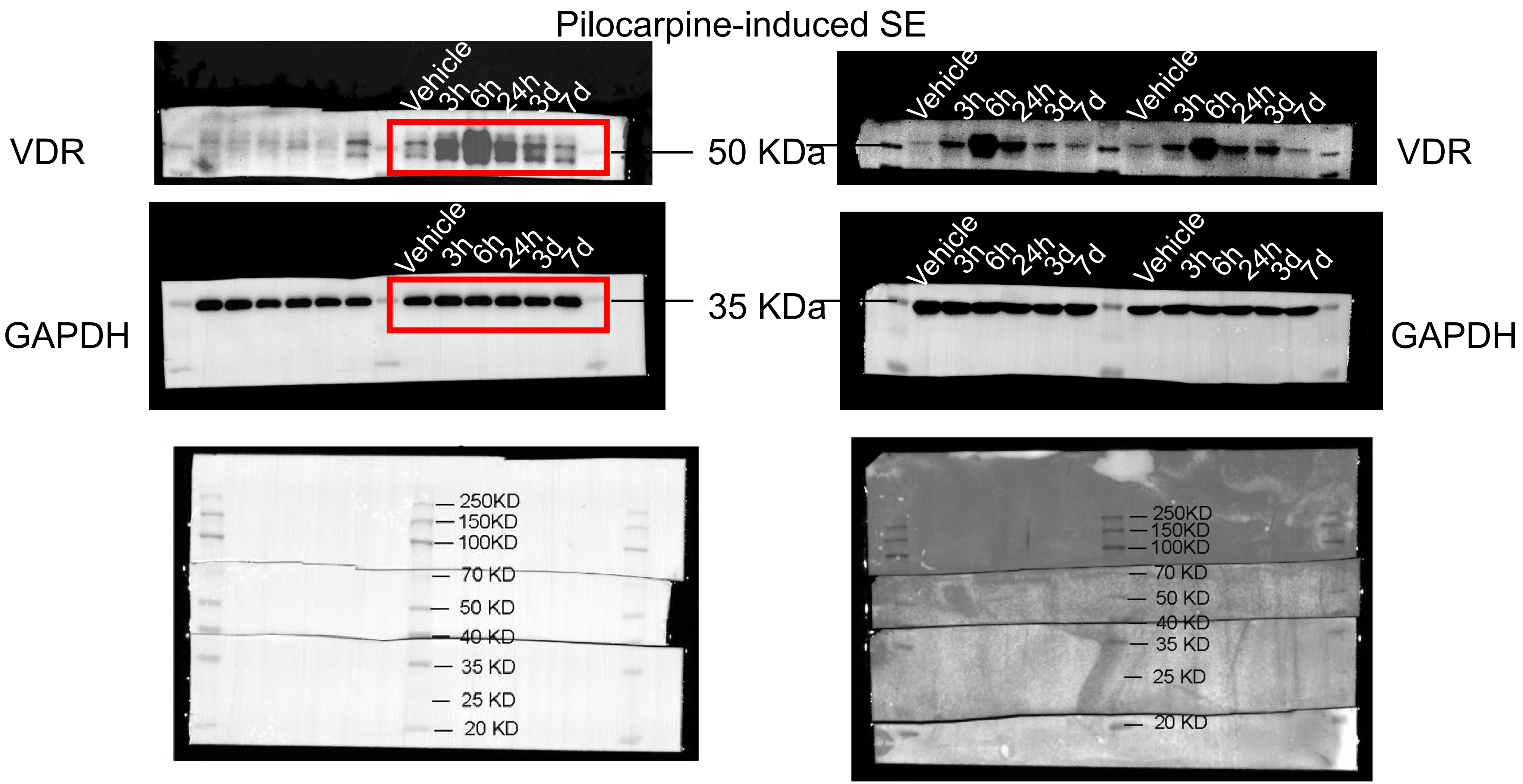

Supplementary Figure 5. Uncropped source blots for VDR analysis in the hippocampus following pilocarpine-induced seizures. GAPDH served as loading control. Rectangles in red indicate representative images shown in Figure 1F.
